# Considerations in setting up and conducting epidemiologic studies of cancer in middle- and low-income countries: the experience of a case–control study of inflammatory breast cancer in North Africa in the past 10 years

**DOI:** 10.1002/cam4.36

**Published:** 2012-10-11

**Authors:** Amr S Soliman, Catherine Schairer

**Affiliations:** 1 Department of Epidemiology, University of Michigan School of Public HealthAnn Arbor, Michigan, 48109-2029; 2 Division of Cancer Epidemiology and Genetics, National Cancer InstituteBethesda, Maryland, 20852-7234

**Keywords:** Breast cancer, Egypt, epidemiology, field methods, Morocco, North Africa, Tunisia

## Abstract

This article illustrates some issues we faced during our experience in conducting an epidemiologic case–control study of inflammatory breast cancer in North Africa. We expect that some of the questions we had to ask in order to address these issues might be helpful to others in setting up epidemiologic studies in developing regions. We describe our experience from different angles including the use of multiple sites to achieve adequate sample size, standardizing diagnosis of disease, identifying cancer cases at the time of diagnosis, control selection procedures, logistics of study implementation, questionnaire development and interviewing, biologic specimens, and procedures for protection of human subjects. We have developed a brief checklist to summarize important issues for conducting future epidemiologic studies in these or similar low- or middle-income countries.

## Introduction

Designing and conducting epidemiologic studies in developing countries can be very challenging due to lack of research and medical infrastructures at study centers, including personnel trained in epidemiology. In this manuscript, we share our experience in designing and conducting an ongoing epidemiologic case–control study of inflammatory breast cancer (IBC) in North Africa that included a molecular epidemiology component.

For background, IBC is a rare, aggressive form of breast cancer characterized by the rapid clinical appearance of erythema (redness), edema, and peau d'orange of the breast. Several studies have reported that IBC constitutes a larger proportion of breast cancers in North Africa than in the United States [1, 2], although the proportion of IBC in some parts of Tunisia appears to be declining [3]. This is why we chose to do the case–control study in Egypt, Tunisia, and Morocco. Our study includes IBC cases, a sample of non-IBC cases, and visitor controls. We collected information via questionnaires, medical record review, clinical examination, anthropometric measurements, saliva samples, tumor tissue, and digital photographs of the breast for the cases and questionnaire, anthropometric measurements, and saliva for the controls.

Our aim in this study is not to describe the specifics of the ongoing study on IBC, but rather to focus on issues that we faced in mounting this low-budget, low-technology study that might be pertinent to other epidemiologic studies in developing regions. These issues include (1) enrolling study subjects at multiple study sites to achieve adequate sample size; (2) standardizing the diagnosis of a disease that has a clinical component; (3) identifying study subjects with a specific disease before treatment is initiated from among many patients seen at study hospitals; (4) sampling cancers as a comparison group; (5) control selection procedures when cases may not be population-based and when hospital-based controls are not feasible; (6) logistics of study implementation in a low-technology setting; (7) development of questionnaires that are relevant to the study population; (8) collection of biologic specimens, including fixation procedures; (9) data management issues; and (10) issues regarding protection of human subjects. We also provide guidelines for determining whether other settings are feasible for conducting epidemiologic studies.

## Methods

### Selection of study sites to accrue a sufficient sample size

Because IBC is rare, we needed to include a number of study hospitals and accrue patients over a relatively long period of time. This posed challenges with regard to standardization of study procedures over both space and time. We selected study sites for the case–control study based on existing relationships with investigators at various study sites, infrastructure for patient recruitment and conducting clinical and epidemiologic studies [4–9], interest among study clinicians in collaborating, availability of a relatively large number of cases to obtain reasonable sample size, and variety of patient backgrounds. To achieve maximum sample size, we selected large referral cancer centers, such as the National Cancer Institute (NCI) – Cairo in Egypt, the Salah Azaiz Cancer Institute in Tunisia, and the Ibn Rochd Oncology Center in Morocco. NCI-Cairo's patient population comes from all parts of Egypt with approximately 45% of patients from Cairo metropolitan area, 30% from the Delta region, and 25% from South Egypt [10]. The Salah Azaiz Cancer Institute's patient population comes mainly from the Tunis metropolitan area and North Tunisia, but patients from central and southern parts of Tunisia also come for diagnosis and treatment [11, 12]. The Ibn Rochd Oncolgy Center is a referral center for patients from the Southern half of Morocco and is home of the population-based cancer registry of Morocco [13]. In all, we included three cancer centers in Egypt, one cancer center in Tunisia, one cancer center in Morocco, and one university hospital in Morocco.

Increasing number of participating hospitals in each country required adjustment of the study procedures to suit each hospital situation without compromising the integrity of the study. For instance, case and control identification procedures had to be modified for each study hospital (to be described later). Ideally, it is logistically easiest to recruit study subjects from as few study hospitals as possible to achieve sufficient sample size.

Investigators who intend to initiate a new study in middle- and low-income countries without prior contacts at study hospitals can research existing literature to identify centers with research experience. Some major cancer centers provide information on websites (e.g., http://www.nci.edu.e.g for the NCI-Cairo). Furthermore, new investigators can network with investigators who were funded to conduct studies in developing countries by searching databases such as the Reporter website of National Institute of Health (NIH) (http://projectreporter.nih.gov/reporter.cfm) or ongoing studies or publications of the International Agency for Research on Cancer (http://www.iarc.fr/).

### Methods to standardize the diagnosis of IBC, a disease whose diagnosis has a clinical component

IBC is largely a clinical diagnosis, although confirmation of cancer by pathologic evidence is also required. Standardization of clinical diagnoses, which are inherently somewhat subjective, across study hospitals and countries can be challenging. The procedures we describe below for IBC may be helpful in the standardization of diagnoses of other diseases with a clinical component. With regard to IBC, we wanted to capture and standardize the degree of redness, edema, and peau d'orange of the breast leading to a diagnosis of IBC. We also wanted to distinguish between IBC and other forms of locally advanced breast cancer. To standardize the clinical diagnosis, we first conducted a pilot study in which cases determined to be IBC at two of the study hospitals were enrolled over a 7- to 9-month period and asked to consent to digital photographs of the breast and detailed clinical examinations in which the presence, extent, and duration of various signs/symptoms of IBC were recorded. We then established several layers of review, first at the study hospitals by senior clinicians and then by two oncologists in the United States, who reviewed the photographs and clinical information. We used the results of the pilot study to establish specific guidelines for the case–control study and to train collaborators on the inclusion criteria for the case–control study. We continued the collection of digital photographs of the breast and detailed clinical information during the case–control study. Digital photographs are uploaded to the study portal (which is an online document publishing system and collaborative software tool that is password protected) or to a CD. Clinical information and digital photographs of the breast are then entered into the NIH National Library of Medicine Interaction Tool for review by outside experts. Each reviewer performs the evaluations through the Internet using a unique username and password. The results of their evaluations are saved in a central database.

### Selecting IBC cases at the time of diagnosis –issues with selecting participants with one disease from many seen at study hospitals

In many major hospitals in developing countries, including our study cancer centers, clinics are busy with large numbers of patients. This number could be in the range of hundreds of patients/day [14–16].

Our challenge was to identify a rare cancer type (IBC) before treatment from among many breast cancer cases and other types of cancers. This challenge is not unique to the study of IBC, but would be present at most hospitals that do not have specific clinics for certain types of cancers/diseases. We found it critical to understand the patient flow at each study hospital. Thus, we sought to determine (1) the department in which suspected breast cancer cases were first seen; (2) whether suspected breast cancer cases were seen in a different clinic/room in that department than previously diagnosed cases; (3) in which department/room breast cancer cases diagnosed elsewhere were first seen; (4) how newly diagnosed cases were referred for treatment; (5) the number and schedules of physicians in the surgery and medical oncology departments; (6) the level of cooperation between the surgery and medical oncology departments; (7) whether free-of-charge patients, those with insurance, or those paying out of pocket were seen in the same clinics; (8) whether the hospital had a central review committee that evaluated all new breast cancer cases; (9) whether logs (paper or electronic) of patients, including diagnosis, age, and residence, were kept in the surgery and medical oncology departments; (10) whether senior physicians worked on a full-time basis or divided their time between their government or University hospital work and private practice; (11) whether the same residents staffed the clinics every day; (12) whether treatment was done on an inpatient/outpatient basis; and (13) the length of time between diagnosis and the start of treatment.

We found this evaluation absolutely necessary and adapted our methods for identifying IBC cases to the existing procedures at each study hospital. For instance, at study hospitals that had a central review committee, we identified a study collaborator who attended the meetings to identify IBC cases. For study hospitals that did not have a central review committee, we followed two procedures: (1) we identified study collaborators who were available in the clinics for enrollment of IBC cases and (2) we asked study collaborators to notify clinic physicians who were not collaborators to refer IBC cases to study collaborators. We also sought to have multiple points of referral of IBC cases to study collaborators (e.g., from both the Surgery Department and the Medical Oncology Department).

We found it particularly helpful to include junior physicians in the study, particularly at institutions that had little rotation among the junior physicians. Linking the study objectives to the physicians' theses and dissertation topics and providing educational opportunities for them such as attending scientific conferences in North Africa or the United States motivated them to continue their contributions to the study.

We found the following issues particularly challenging with regard to enrollment of all eligible cases: (1) adequate backup duration absences of study collaborators due to vacations, meetings, illness, or periods of study for examinations; (2) short rotations in the medical oncology departments for junior physicians at certain study sites; (3) the heavy schedule of senior study collaborators; and (4) changing hospital procedures that would interfere with study procedures for accrual. Therefore, we highly recommend that any study have procedures for review of medical records to ascertain the completeness of case accrual.

### Selection of non-IBC cases – methods for sampling comparison cases

In some types of studies, one may want to sample a certain type of cancer rather than recruit every case. This was the case for the non-IBC comparison group in our IBC study. With regard to comparison non-IBC breast cancers, the challenge was to select a sample from large pools of cases. Our methods for selecting non-IBC cases were also based on the careful evaluation of hospital procedures that we described above and varied depending on the hospital. We used logs kept by the surgery and medical oncology departments when available to identify non-IBC cases matching the IBC case within 5-year age groups and broad geographic area of residence. In other hospitals, we selected non-IBC cases from cases reviewed at weekly committee meetings. Finally, at some study sites, we randomly selected clinics from which to find matching non-IBC cases. To do this, we assigned a number to each clinic from which non-IBC cases could be selected and then used a simple SAS random number algorithm to randomize the assigned clinic numbers to determine from which clinic each non-IBC case was to be selected. If more breast cancer cases were seen at one clinic compared with another (e.g., if 80% of patients at a hospital were seen in a “free” clinic and 20% of patients in an “insurance/private” clinic), we assigned numbers to the clinics in proportion to the number of cases they saw. Depending on the level of cooperation between the surgery and medical oncology departments, we selected non-IBC cases before or after mastectomy.

The determination of the clinic from which each non-IBC case was to be selected was done at the NCI-USA and was communicated to the study collaborators as described in the section “Logistics of study implementation.” We ascertained from questions on our forms whether collaborators were adhering to the randomization scheme.

### Controls

We did not select population-based controls because we were not assured that our cases were population based. Furthermore, it would not have been feasible to select population-based controls because such things as telephone use and driver's license registration are very incomplete in the areas where our study is being conducted. We also faced additional challenges in recruiting controls. We could not recruit noncancer controls from our study patient populations because most sites were cancer centers. Noncancer hospital-based controls were not feasible because we would have had to enlist the cooperation of nearby general hospitals, thereby greatly increasing the complexity of the study. Therefore, we chose to select controls from those visiting certain types of other cancer patients at our study hospitals, as has been done in other studies [17–21]. To do this, we evaluated at each study hospital (1) the number of inpatient surgical and medical oncology wards, the number of rooms in each ward, and the number of beds in each room; (2) whether there were separate inpatient wards for free-of-charge patients, those with insurance, or those paying out of pocket; (3) whether there were inpatient wards for patients coming from a distance; (4) visiting days and hours; (5) whether there was a charge for visiting; (6) methods for ascertaining the diagnosis for the patients in each hospital bed; and (7) availability of study staff to complete study procedures, including interviews, during visiting hours. Based on this information, we developed randomized procedures to choose the wards from which to choose noncancer visitors of other cancer cases. We used simple randomization schemes similar to those we used for randomly selecting clinics for identifying non-IBC cases. For instance, we assigned a number to each ward from which visitors could be selected, then used a simple SAS random number algorithm to randomize the assigned ward numbers to determine from which ward each visitor was to be selected. We attempted to make the visitor selection reflective of the patient population by assigning multiple numbers to types of wards with the largest patient population (e.g., if 80% of the patient population was in “free” wards vs. 20% in “insurance/private” wards, we assigned 80% of the random numbers to the “free” wards and “20% to the “insurance/private” wards). We designed our forms to ascertain whether the randomization schemes were being followed. The determination of the ward from which each visitor was to be selected was done at the NCI-USA and was communicated to the study collaborators as described in the section “Logistics of study implementation.”

Particular challenges with selection of controls included (1) finding interviewers who were available during visiting hours; (2) finding visitors meeting the matching criteria for age and geographic residence; and (3) convincing the study collaborators that recruiting controls was as important as recruiting cases. Many clinicians have experience with research that involves cases but no controls.

### Logistics of study implementation

As noted previously, our study was low-budget and therefore, necessarily low-technology. Therefore, we used simple methods for study implementation. As noted previously, the hospitals that participated in this study saw a high volume of patients and were crowded and busy. In this setting, management of study documents to ensure completion of all required steps and to minimize loss of study materials is very important. Thus, we included all study materials for each study subject in an inexpensive 1-inch three-ring plastic binder with study documents in the order in which they were to be administered and dividers indicating who was to administer the study documents. Instructions were placed at the beginning of the binder. Forms included selection forms for visitor controls, eligibility forms for IBC and non-IBC cases, consent forms, the questionnaire, clinical examination forms, medical record review forms, pathology report forms, instructions for saliva collection and anthropometric measurements, and taking digital photographs. We designed selection and eligibility forms to record nonresponse, which is crucial to report for publication and for understanding whether the subjects enrolled in the study reflect the populations of interest. For instance, if visitors were eligible but refused, this was recorded on the visitor selection form. If IBC or non-IBC cases were eligible, but refused, this was recorded on the eligibility forms. A plastic pencil case was placed at the beginning of the binder to hold the vials for saliva collection. Labels identifying the study hospital, the type of study subject, the study ID number, the age category, and broad geographic area of residence for matching were placed on the cover of the binders. For non-IBC cases and visitors, the clinic or ward from which the study subject was to be selected was also identified on a label placed on the cover of the binder. Each subject type (IBC, non-IBC, visitor control) had a different color binder. These binders were assembled in the United States and shipped to study hospitals. To facilitate the shipping of the binders, we completed an NIH 1884-1 Commercial Invoice form that allowed delivery of the binders directly to the study hospitals. Without this form, study collaborators had to pick up the binders at the customs office. On occasion, we have had some difficulty getting the saliva vials through customs in the collaborating countries. In fact, at some sites it became necessary to hand carry saliva vials during travel by U.S. study collaborators to avoid problems with customs.

With regard to response rates, particular challenges included persuading clinicians that it was important to record nonresponse. It was also challenging to design forms that recorded nonresponse of eligible subjects but not nonresponse of ineligible study subjects.

Data keying was done at the study sites using Viking Data Keying Software, a straightforward, well-documented commercial system. The double-keyed data files are uploaded to the study portal at the study sites and are then downloaded from the portal and merged into SAS datasets in the United States.

In addition to being used for posting keyed data files and photographs, the study portal is also used to post all the documents for the study and to provide links for Institutional Review Board (IRB) and Federal-Wide Assurance (FWA) Registration.

## Questionnaires and Interviewing

The design of clinical forms and medical review forms was developed in collaboration with the local clinicians in each site. The clinicians' input was extremely helpful in developing user-friendly forms that capture all the needed clinical information.

Design of the epidemiologic questionnaire was challenging because the study is in three countries, with patients coming from both rural and urban areas. To accurately capture information related to socioeconomic status, education, occupation, contraception, childbearing, and breastfeeding in the different study populations, we talked extensively to study collaborators and used data from national surveys and other publications to help in determining questionnaire content. Important resources in questionnaire development included the following: (1) Egypt demographic and health survey 2005 [22]; (2) Socioeconomic differences in Health, Nutrition, and Population – Egypt 2000 and Morocco (2003–2004) compiled by the Health, Nutrition, and Population (HNP) Family of the World Bank's Human Development Network [23]; (3) Measuring women's work in developing countries [24]; (4) Economic and social conditions in North Africa – Part III – the economic participation of women in North Africa [25]; (5) women's time use survey in Morocco [26]. We also found information on the distribution of smoking, obesity, female literacy, contraceptive use, and consanguinity from other sources [27–34]. We also researched the leading crops grown in each country and the types of pesticides most commonly used.

Cultural sensitivity in developing the questionnaires, setting up the order of questions, and developing questions for sensitive issues was very important. For example, we learned that we should not start the questionnaire by asking about some demographic information of patients. We were told that starting the epidemiologic questionnaire with the demographic section will sound like a “police interrogation” of subjects. In our study, we included address, contact information, or relatives to be contacted in the future, if needed, age, place of birth, and marital history in the beginning but household conditions and appliances farther back in the questionnaire. It should be noted that the order of questions is culture specific, and it should be considered based on the local culture, preference, and settings in each country or region. As an example of a culturally sensitive topic, we were interested in including questions about subjects' exposure to mice in the household environment. We were advised by the local clinicians to tailor the sets of questions on this topic in an indirect way because it would be considered insulting to ask about mice in the household. We were advised to begin by first asking about subjects' knowledge of mice control campaigns in their neighborhood, followed by asking about their knowledge of mice in neighbors' homes, and finally asking about their use of rodenticides for killing mice in their homes. We also tried to be culturally sensitive to perceptions about cancer. For instance, local clinicians told us that some women refer to breast cancer as the “bad” disease of the breast, call malignant tumors “female” and benign tumors “male,” and refer to cancer hospitals as “bad” hospitals. We pilot tested the questionnaire with employees of the study hospitals from a variety of backgrounds.

Study subjects were administered the questionnaires by interviewers. Although patients and physicians in the Egypt, Tunisia, and Morocco all speak Arabic, different dialects of Arabic are spoken in each country [2]. Some Tunisians and Moroccans also speak French, but others do not. Thus, we translated the questionnaire into the spoken Arabic dialects for each country and also into French.

The final version of the questionnaire included the following sections in this order: contact information; age; religion; ethnic background; childbirth history; educational background of study subject; marital status; husband's educational and occupational background; menstrual history; contraceptive history, infertility, and menopausal hormonal use; breastfeeding history; medical history; family history of breast cancer; agricultural history; other occupational history; anthropometry; exposure to tobacco smoke; residential history and household appliances as surrogates for income; access to health care; level of cooperation in the interview; and current body measurements. The order of the above-listed sections of the questionnaire yielded the best flow of delivering the questions and cooperation of study subjects as shown in the pilot testing of questionnaires.

For some study subjects, the questionnaire, which on average took 45–60 min, seemed long and interviewers had to give them breaks in order to complete the entire questionnaire. Some patients have difficulty in recalling age at childbirth, age at starting and stopping contraceptive use, age of starting different agricultural occupations, and exact weight at different ages. To help recall of ages at childbirth, we also obtained information on the current ages of the children, so that we could subtract the current age of the children from the study subject's current age to obtain the study subject's age at the birth of each of her children. The interviewers reminded the subjects of their childbirth history to help them recall their periods of contraceptive use. They used age of marriage and other major life events to help them recall ages of different agricultural and other occupations. With regard to weight, the questionnaire also included a diagram of body types at different ages, which the study subjects were able to complete. We also measured current body weight, height, and hip, waist, and chest circumference. Gender of the interviewer was also important. In one study hospital, the only interviewer available was male, and he was uncomfortable asking about bra size and taking chest and hip measurements. To compensate for this, the study clinician asked these questions and took the measurements.

Medical examination and review forms were in English in Egypt because English is the medical language of medical education. French was the language of the same forms in Tunisia and Morocco. Translation of questionnaires into spoken Arabic dialects was challenging because most professional translators are comfortable with translation into standard Arabic, not into spoken colloquial Arabic. This resulted in considerable review and revision and back-translation of the translated documents. A possible alternative would have been to have the questionnaires in standard Arabic and to have the interviewers translate orally into spoken Arabic dialects, but this would have resulted in less standardization of the spoken dialects.

Consenting is also a subject that should be considered with care in developing countries. The reasons include the large percent of illiterate patients who cannot read and sign the consent form. In addition, because of the need for free medical care, some patients may have the impression that they might not receive medical care if they do not participate in research studies. We had to be very explicit in the wording of the consent forms to avoid this possible impression. We had to have a witness sign the consent form after its verbal reading to each study participant who could not read or write. Witnesses were typically family members and that facilitated the consenting process and the high response rate of participation in the study. On some occasions, the husbands of potential study subjects refused to allow their wives to participate in the study even though the wives wanted to do so. In other middle- and low-income countries, the effect of the relationship with the witnesses needs to be investigated on consenting and study participation. Patients were asked to consent on each section of the study (e.g., interviewing, providing saliva samples, agreeing to use the tumor tissues in case of IBC and non-IBC, and taking photographs of their breast without showing their face).

## Biologic Specimens

We encountered the following issues in obtaining paraffin-embedded tumor tissue specimens: (1) ensuring that the method of formalin fixation is adequate to obtain viable DNA; (2) obtaining sufficient tumor tissue using the standard diagnostic techniques in the study hospitals; (3) obtaining tumor tissue for cases diagnosed outside the study hospitals; and (4) reluctance of collaborators to share their paraffin blocks for research outside the home country.

We addressed the appropriate method of fixation by providing the standard protocol for preparing paraffin blocks that we used at the University of Texas M. D. Anderson Cancer center in the late 1990s. There were few differences between the M. D. Anderson protocol and the local protocols except that the latter used concentrated formalin for preserving the tissues after resection and possibly long duration (1–2 days) between resection and processing. Our M. D. Anderson protocol used 10% formalin and a processing of specimens within 3–4 h. We were able to persuade the local institutions to follow the U.S. protocol because all it required was dilution of the formalin with water without any expenditure of money or other resources. With the U.S. protocol, we were able to obtain viable tissues that we used in our subsequent studies [6, 12, 35–37].

With regard to obtaining sufficient tumor tissue, it is critical to determine what diagnostic techniques are standard in study hospitals. For instance, diagnosis of breast cancer, including IBC, is frequently done by fine needle aspiration, but this method does not yield sufficient tissue for research purposes. A true cut biopsy is more likely to yield sufficient tumor tissue, although yield might not always be enough and is less than is obtained from mastectomy specimens. Because a true cut biopsy also has advantages in terms of diagnosis, our collaborating clinicians agreed to do true cut biopsies instead of fine needle aspiration biopsies to accommodate the research study.

With regard to obtaining tumor tissue for cases diagnosed outside the study hospitals, we note that it is often customary for patients to bring their slides from the diagnosing hospital or pathology laboratory to the study hospital. Personal communication of the study clinicians and pathologists with the hospital or laboratory where the biopsy was done is extremely important for having a complete set of tissues. Many tissues that were diagnosed outside our collaborating cancer centers were processed at private laboratories of the institutions' pathologists and were prepared according to our study protocols.

It is important to do periodic checks of the quality of DNA from the tissues obtained from the collaborating cancer hospital or private laboratories to ensure high quality. Pathological diagnosis is always confirmed by our collaborating pathologists in North Africa and the United States. Embedding of tissues in cassettes varied between the collaborating institutions and sometimes needed re-embedding in the United States if tissues are directly fixed on paraffin without cassettes. Relying on bar-coding of specimens in developing countries should not be selected as an option because of lack of scanners and computers that would download and read scanned numbers. Instead, it would be useful to scan and print IDs before shipping forms and labeling tissues and slides after they are received in the United States.

Regarding the reluctance of collaborators to share their patients' paraffin blocks outside the country, this was mainly due to country rules on exporting biologic specimens or their interest in local technology transfer to their country. We understood the country restrictions on exporting tissue blocks and instead we obtained cut tissue slides instead of paraffin blocks. When technology transfer was the issue, we followed three approaches: (a) we developed tissue microarrays in the United States and shared duplicates with the local collaborators; (b) we supplied the local collaborators with reagents to perform the laboratory assays locally and compared the quality with the U.S.-generated results from the same specimens; or (c) we provided training to the local collaborators in the United States to return back to their home countries to perform the laboratory assays.

Obtaining saliva samples is being accomplished using Oragene saliva collection kits, which can be stored at room temperature for years, require no special preparation or handling, and are easy to use by study subjects, for the most part. The only precautions for saliva collection are the need for the subject to avoid drinking, eating, or chewing gum for 30 min before spitting and the possible expiration of the reagent included in the saliva tubes for preservation, if the tubes are kept past their shelf life.

Regulations regarding the import of biologic samples into the United States can be found at the following website: http://www.cdc.gov/od/eaipp/faq.htm. Etiologic Agent Import Permits are not required for the transport into the United States of noninfectious materials – for example, formalin-fixed specimens, tissues, or slides, and human or animal diagnostic specimens such as blood, urine, and tissues in which there is no evidence or indication that such material contain an infectious agent. However, the following recommendation is taken directly from the CDC website: CDC advises that importers of materials that do not require a CDC import permit include a signed statement, on their official letterhead, from the person responsible for the shipment of this material with the following information:

- a description of the material;
- a statement that this material meets one of the above criteria (e.g., human urine diagnostic specimens in which there is no evidence that such material contain an etiologic agent); and
- verification that the material has been packaged, labeled, and transported in accordance with all applicable regulations.

## Training, Quality Control, and Data Management

We tried group training of interviewers and study coordinators at one of the collaborating sites in North Africa, but the efficiency of this training was lower than our site visits to individual hospitals and sites. For instance, visa issues and agreeing on one place and time period where the collaborators leave their routine hospital work were limiting factors for the effectiveness of the group training.

Students from the University of Michigan School of Public Health and Cancer Center also help in monitoring the study. They conduct summer research projects funded by the NCI through an R25 educational grant and the University of Michigan research and training grants. The students travel to monitor the study, train local collaborators, and streamline unresolved issues. Involving students was very helpful in facilitating IRB, questionnaire design, testing the questionnaire, and monitoring the study and their research resulted in several publications [2, 36–44]. For instance, one student is reviewing medical records during the period of the study to assess the adequacy of our case accrual methods and reliability of medical records in identifying any missed cases during our study recruitment.

Involvement of students was a useful research experience for them in international epidemiology and facilitated the field study.

In addition, the University of Michigan in collaboration with the Office of International Affairs of NCI has sponsored the University of Michigan Graduate Summer Courses in cancer epidemiology and translation of epidemiology to cancer prevention and control. Several of our collaborating clinicians from North Africa have participated in these courses and the exchange of knowledge and experience improved their engagement in the study and understanding of epidemiologic methods and applications to cancer prevention.

We used simple EXCEL files for study management in the United States and at some study sites. Other sites used paper study management systems because computer access was difficult. Low-technology study management procedures were more successful because of lack of high-efficiency computer systems, available high-speed Internet, and absence of other advanced technological procedures such as wireless Internet connection, remote access of computers, and institutional servers with automatic backups that are increasingly becoming routine in U.S.-based research studies. In fact, home availability of the Internet in Egypt and Tunisia ranges between 25% and 40% [45]. While investigators have high-speed Internet for their personal use at home, government hospitals in North Africa still have limited Internet capabilities. The study has a portal for uploading breast photos and keyed data, but this was sometimes difficult for collaborators to access due to poor Internet connections. It is important to note that with expanding Internet technology in developing countries, there are new tools for better Internet connection even without relying on the infrastructure of hospitals. Wireless Internet cards, modems, and high-speed Internet from cell phone connections are becoming available in many developing countries.

## Regulatory Requirements

For an international multicenter study like ours, with inclusion of a U.S. Federal Government funding component, the regulatory requirements that had to be met were many. These included approvals from IRB at sites in the United States and in the collaborating countries, FWA registration for all collaborating institutions, and payment (Central Contractual Registration [CCR] and the Online Representations and Certifications Applications [ORCA]). While these approvals and certifications are routine procedures for researchers in the United States, they were a challenging experience for our international collaborators. Issues were (1) lapsed IRB and FWA registrations at some study sites; (2) disbanded IRB committees that had to be reconstituted according to U.S. regulations; (3) complicated registration instructions that were not in the native language of the study collaborators; (4) intimidating legal language; (5) difficulty in remembering passwords and access codes to the electronic websites; (6) poor computer access; (7) signatory officials who were not part of the research team; and (8) registrations lapsing during the course of the study because not all collaborators routinely check e-mails, thus missing notifications that registrations needed to be renewed. We had to walk the collaborators through the certification process through numerous emails and site visits. At some sites, we also found that IRB committees did not meet until they had a sufficient number of protocols to review. Thus, plenty of time should be allowed to accomplish these registrations. U.S. collaborators should make every effort to be cognizant of registration lapse dates. Information on U.S. Government regulations regarding IRBs and FWAs can be found at the following address: http://www.hhs.gov. Ethical guidelines for international studies can also be found in the book “International Ethical Guidelines for Epidemiologic Studies” from the Council for International Organizations of Medical Sciences (CIOMS) studies. We note that the Health Insurance Portability and Accountability Act of 1996 (HIPAA) does not pertain to institutions in low- and middle-income countries.

The system of invoicing to the NCI and reimbursement after provision of the service of recruitment was a new concept for our international collaborators. It is important to note that our payment system was based on recruitment of triplet sets of IBC, non-IBC, and control subjects. This payment system for studies in developing countries is significantly more efficient than payment on percent salary effort or monthly basis as is the case in the United States. Because of low salary levels in developing countries, the percent effort compensation does not work there.

## Other Logistical Considerations

In the current era of global and international health, political circumstances are an integral part of the dynamics of joint research and international collaborations. The changing politics in the Middle East starting from the Iraq war in 2003, which delayed the start of the study, and the revolutions in Egypt and Tunisia in 2011, which slowed the flow of patients to study hospitals due to security concerns, have had their impact on the pace of the research study. In fact, we had to extend our contracts to the study sites to account for accrual over a longer period of time.

Other logistical points to consider include understanding the hierarchy and internal politics of the collaborating institutions, the importance of inclusion of senior, mid-career, and junior collaborators, understanding the system of retirement for senior collaborators, and finding opportunities for motivating clinicians to collaborate on an epidemiologic study by including aspects related to clinical research, financial incentives, and other opportunities for professional growth, such as authorship on manuscripts or training in epidemiology. For instance, some of our clinician collaborators were interested in measuring breast size from mammograms, so we incorporated questions about this in our medical record review form. Other clinician collaborators are participating in a clinical trial of IBC during the same period of time as the epidemiologic study, which increased interest and motivation. We further funded training of several collaborators at short epidemiology courses conducted in the Mideast by the first author of this article.

## Important Efforts Not Successfully Undertaken

We attempted to measure breast size in two ways: by obtaining information on bra size on the questionnaire and by recording certain measurements from the mammograms (vertical, horizontal, and diagonal dimensions of the breast). However, our efforts were not entirely successful because women living in rural areas frequently do not wear bras. Moreover, the physicians were not used to making the measurements on the mammograms so these measurements were rarely made. Successful implementation of the mammographic approach would require more extensive training than we did. Breast immersion and measuring the volume of water replaced were impractical in a routine hospital setting. Developing new feasible and practical methods for estimating breast size would be important for epidemiologic studies of breast cancer considering the wide range of variation in breast size between populations.

Our study would have benefited from more frequent and longer site visits by U.S. principal investigators. In general, we had funding to do two site visits per year, with 1–3 days at each study site. It would have been helpful to have four site visits per year, with a minimum of 2 days at each site. Students were funded for a period of 3–4 months, the limit allowed by the training grant and their class schedules. Additional funding for more extended stays and more flexible and tailored curriculum of students would have enabled them to stay longer at each site.

Regular use of advanced tools of electronic communication with the collaborators in North Africa would be helpful. Currently, there are software programs such as Adobe Connect that enable verbal and visual communication and sharing and editing of documents between collaborators at different international locations. Use of these techniques would enhance effective communication at limited cost.

Diagnostic criteria for cancers other than IBC, such as hematologic malignancies, may differ among countries. Therefore, teaching sessions by expert pathologists should be included in the studies of such cancers in order to minimize diagnostic differences among study sites. Studies of cancers that require diagnostic imaging (e.g., liver and pancreatic cancers) might require upgrading the infrastructure of the recruiting hospital and physician training to capture the cases.

We have summarized our recommendations for the planning and conduct of epidemiologic studies in low- and middle-income countries in a checklist (Table 1).

**Table 1:** Checklist of critical elements for assessing feasibility of an epidemiologic case–control study at a site in low- and middle-income countries

(1) Study site(s) has willing collaborators and qualified staff to conduct the study.
(2) A sufficient number of cases can be accrued.
(3) Cases can be identified at or near the time of diagnosis.
(4) Medical records with patient and clinical information are available at study site.
(5) Biologic specimens that are sufficient and suitable for research purposes can be collected.
(6) Study hospital is willing to release biologic specimens for research.
(7) A suitable control group can be found (either at study hospital, affiliated or neighboring hospital, from the neighborhood or population based).
(8) Protection of human subjects is adequate.
Checklist for critical elements for study implementation
(1) Detailed scientific protocol.
(2) Adequate funding.
(3) Development of study forms and documents.
(4) Pretesting of forms and documents, including questionnaire.
(5) Training of study personnel.
(6) Quality control procedures.
(7) Adequate provisions for study management and data keying.
(8) Permits for obtaining and shipping biologic specimens.
(9) Obtain clearances related to protection of human subjects.
Checklist for desirable elements
(1) Develop sustainable infrastructure for future studies.
(2) Train personnel for future studies.

## Discussion

Our experience in conducting this epidemiologic study in North Africa highlights the importance of the some general points for those interested in future epidemiologic case–control studies in North Africa and other middle- or low-income developing countries.

1. It is very important to understand the healthcare system of the country and the cultural and behavioral factors related to seeking medical care. This includes understanding the referral patterns of cases to study hospitals. This information is crucial for efficient recruitment, selection of an appropriate control group, and generalization of the results. For instance, if many cases of the disease of interest never seek health care, it is not recommended that population-based controls be used.
2. In selecting a control group, it is also very important to understand the culture of the country. For instance, if telephone use and driver's license registration are very incomplete in the areas where the study is going to be conducted, certain methods for selecting controls, such as random digit dialing, are not recommended.
3. Defining the precise clinical and/or pathological nature of the disease to be studied is essential and may vary by the cancer site/type to be studied. For example, precise clinical diagnosis is the most important diagnostic element for IBC. On the other hand, precise pathological and/or immunological diagnosis is the most important element for inclusion of lymphoma cases in epidemiologic studies.
4. Epidemiologic studies should consider the inclusion of clinical components and/or coordinate with clinical trials of the same disease that might be underway in study hospitals. This point is especially important because of the limited number of trained cancer epidemiologists in developing countries and the leadership of research studies by clinicians. In the meantime, training and creation of a new cadre of trained cancer epidemiologists is also important.
5. Readiness for unexpected changes in the pace of the study due to political, personnel, or hospital setting changes is crucial. Alternative plans should be considered and be available for continuation of the study under new circumstances.
6. It is important to explore all avenues for funding and training of junior clinicians, study managers, and interviewers. Sending collaborators to short courses in cancer epidemiology, offering training options in the United States, and inclusion of students from the United States into the study are very helpful. Coupling of local and U.S. students can also add many benefits to the progress and quality control of the study.
7. It is also important to appreciate technical difficulties, busy clinics, and overloaded local systems for patient care in developing countries. It is important to consider reliance on low-technology methods for data management, quality checking, and monitoring of the study rather than high-technology sophisticated software system that require high-speed Internet and other technology that might not be regularly available in hospital settings.
8. It is important to know that there are likely unwritten cultural codes in middle- and low-income countries and their hospitals. For instance, internal conflicts between departments and fear of displeasing mentors or powerful institutional leaders may constitute unwritten codes that hinder the progress of the study. It should be noted that understanding such conflicts may need time and close engagement with the collaborators. In some middle- and low-income countries, the primary income of cancer center faculty will be earned in their private practice. Sometimes, their service at the university will be unpaid. Consequently, university department chairs, deans, and administrators might have little power to influence their faculty members, and this can come as a surprise to researchers from high-income countries. Therefore, it is important to consider whether a particular collaborator should be compensated with financial incentives separately from the grant budget that is allocated for the cancer center or through other means. Private discussions must resolve these matters. Other unwritten codes include the difficulty and misunderstanding of spoken English. Furthermore, “yes” does not always means “yes” and “no” does not always mean “no.” There are also many cultural barriers that may prevent collaborators from expressing their disagreement. For example, researchers and clinicians may feel embarrassed to inform their collaborators from high-income countries of their disagreement about a study procedure or feasibility of success of a study procedure. The result will be a false impression of agreement while failure of achievement will be the outcome. On the other hand, researchers and clinicians may show rejection of certain study procedures or techniques because of their lack of knowledge of this procedure or its implementation. Because they may feel that they may be displeasing the collaborators from high-income countries if they admit so, they may reject the procedure rather than learning about it.

We expect that there will be many overlapping points between our experience on the epidemiology of IBC in North Africa and other research studies on breast or other cancers in other middle- or low-income developing countries. Possible variations between our experience and future research studies include the level of development of the country, study design, cancer site, cultural and behavioral factors of populations, access to medical care, referral systems of patients, and the medical education of the country. While we attempted to maximize the success of our study using the methods described in this article, other study- and region-specific measures may be tailored according to the aims and local circumstances of the study setting to enhance opportunities of success.
